# Isn’t it ironic? Neural Correlates of Irony Comprehension in Schizophrenia

**DOI:** 10.1371/journal.pone.0074224

**Published:** 2013-09-10

**Authors:** Alexander M. Rapp, Karin Langohr, Dorothee E. Mutschler, Stefan Klingberg, Barbara Wild, Michael Erb

**Affiliations:** 1 Department of Psychiatry, University of Tuebingen, Tuebingen, Germany; 2 Department of Neurology, Marienhospital Stuttgart, Stuttgart, Germany; 3 Biomedical Magnetic Resonance, Department of Radiology, University of Tuebingen, Tuebingen, Germany; Hangzhou Normal University, China

## Abstract

Ironic remarks are frequent in everyday language and represent an important form of social cognition. Increasing evidence indicates a deficit in comprehension in schizophrenia. Several models for defective comprehension have been proposed, including possible roles of the medial prefrontal lobe, default mode network, inferior frontal gyri, mirror neurons, right cerebral hemisphere and a possible mediating role of schizotypal personality traits. We investigated the neural correlates of irony comprehension in schizophrenia by using event-related functional magnetic resonance imaging (fMRI). In a prosody-free reading paradigm, 15 female patients with schizophrenia and 15 healthy female controls silently read ironic and literal text vignettes during fMRI. Each text vignette ended in either an ironic (n = 22) or literal (n = 22) statement. Ironic and literal text vignettes were matched for word frequency, length, grammatical complexity, and syntax. After fMRI, the subjects performed an off-line test to detect error rate. In this test, the subjects indicated by button press whether the target sentence has ironic, literal, or meaningless content. Schizotypal personality traits were assessed using the German version of the schizotypal personality questionnaire (SPQ). Patients with schizophrenia made significantly more errors than did the controls (correct answers, 85.3% vs. 96.3%) on a behavioural level. Patients showed attenuated blood oxygen level-dependent (BOLD) response during irony comprehension mainly in right hemisphere temporal regions (ironic>literal contrast) and in posterior medial prefrontal and left anterior insula regions (for ironic>visual baseline, but not for literal>visual baseline). In patients with schizophrenia, the parahippocampal gyrus showed increased activation. Across all subjects, BOLD response in the medial prefrontal area was negatively correlated with the SPQ score. These results highlight the role of the posterior medial prefrontal and right temporal regions in defective irony comprehension in schizophrenia and the mediating role of schizotypal personality traits.

## Introduction

Ironic remarks, although common, represent a comprehension challenge for the listener. In the case of linguistic irony, what is said is, in most cases, the exact opposite of what is intended (e.g., ‘oh brilliant’ when something bad happens). Several factors make research on comprehending ironic and sarcastic remarks in schizophrenia very interesting. Irony, alone, is interesting because it is so frequently used in everyday language, as indicated by linguistic analyses [1], [2], [3]. Understanding irony relates to ambiguity resolution [4]. Irony is ambiguous *per se* and is often used in difficult stages of communication [5], [6], [7], [8]. Interaction deficits, defective appraisal of the intentions of others, and language abnormalities, particularly in an ambiguous context, are hallmark features of the psychopathology of schizophrenia.

Comprehension of ironic remarks in schizophrenia was recently the topic of a series of studies [9], [10]. It is now confirmed that patients with schizophrenia have deficits in comprehending ironic remarks [11], [12], [13], [14], although not all patients exhibit the deficit [15], [16]. It is generally assumed that the difficulties in irony comprehension in patients with schizophrenia have a common neural basis. However, little is known about the neural correlates of irony comprehension in schizophrenia. Unravelling these neural correlates is important because irony comprehension tasks could possibly serve as a window into the dysfunction of several neuronal subsystems in schizophrenia. For instance, irony comprehension relates to hemispheric interaction. In essence, irony comprehension involves both the cerebral hemispheres and their interplay [17], [18], [19], [20], [21], [22], so that irony comprehension may provide insight into hemispheric interaction in schizophrenia [23], [24]. Another functional system that might relate to defective irony comprehension in schizophrenia is the mirror neuron system. It has been proposed that mirror neuron dysfunction may underlie the social cognition deficits in patients with schizophrenia [25], [26], [27]. Moreover, this system has been reported to be involved in the comprehension of irony (see [19]). A third possibly affected system is the brain default mode network, which is altered in schizophrenia [28]. The default mode network is involved in theory of the mind (TOM; [29]), which is also an important cognitive operation in irony comprehension [30], [31]. The medial prefrontal cortex, a key region for the comprehension of ironic stimuli in healthy subjects [19], [32], [33], [34], is part of this network. It has been previously demonstrated that medial prefrontal cortex dysfunction may underlie impaired affective mentalizing in schizophrenia [35], [36], [37].

Several theoretical models have been proposed for brain dysfunction during irony comprehension in schizophrenia. Perhaps, the most commonly proposed model is that the defective irony and sarcasm (that is, irony with hurtful intentions) comprehension in individuals with schizophrenia is due to deficits in TOM and perspective taking [9], [38]. Indeed, TOM is one of a number of essential cognitive steps required for irony comprehension [31], [39]. This model assumes that the TOM is the key problem and implies that the functional neuroanatomical deficits are present in brain regions crucial for TOM, such as the medial prefrontal cortex.

Misinterpretation of ironic remarks may also contribute to developing or worsening positive symptoms such as persecutory delusions. The cognitive model proposed by Salvatore et al. [27] assumes misinterpretation of “ambigous and hard-to-interpret communicative signals” such as “ironic comments” in co-occurence with the factors above may induce delusions. Thus, investigating the (mis)understanding of ironic remarks in schizophrenia will provide insights into an important aspect of schizophrenic psychopathology.

Leitman and colleagues [40] proposed a model that deficits in prosody and speech melody perception are important for sarcasm detection deficits in schizophrenia. This model suggests a deficit in the brain regions associated with comprehension of prosody, such as the right superior temporal cortex [41], [42]. Further, this would mirror fMRI findings in autism, where prosody interacts with fMRI correlates during irony comprehension [43]. Nevertheless, the importance of prosody for irony comprehension is controversial because it is only one of a number of markers for irony [44]. Irony without speech melody is not only possible [45], but is actually very frequent in written language [2], [3], [46]. In fact, patients with schizophrenia show abnormalities in tasks with written irony [15], [16].

Another model states that dysfunction of the right cerebral hemisphere and/or defective interaction between the cerebral hemispheres may underlie difficulties in deciphering irony and sarcasm in schizophrenia [23], [47]. Traditionally, comprehension of non-literal stimuli is ascribed to the right cerebral hemisphere ([48]; see [20], [49] for a critical discussion). Indeed, there is sufficient evidence that the right hemisphere is involved in the comprehension of irony. Both the left and right hemispheres are more involved in comprehension of ironic remarks than in lower linguistic functions [18], [22], [33]. The hypothesis that the comprehension difficulties in schizophrenia represent a right hemisphere deficit is, therefore, plausible, but currently lacks experimental support. The functional lateralisation of irony comprehension in schizophrenia is also interesting from another point of view; increasing evidence indicates that language lateralisation is reduced in schizophrenia [50], i.e., in schizophrenia, language is shifted to the right cerebral hemisphere to a higher extent. It has been proposed that language functions previously outperformed by the right hemisphere might, in a similar manner, shift to the left hemisphere [23], particularly in patients with severe thought disorder [24], [47], [51]. The latter assumption is supported by findings of studies in patients with severe thought disorders and language production tasks [52], [53], and in patients with difficulties in literal language comprehension [54], [55], [56].

Dysfunction of the brain language system is crucial for another model of disturbed irony comprehension that was recently proposed by our group [19], [57], in which we assumed a role of the frontotemporal language semantic comprehension network for disturbed irony comprehension in schizophrenia and adopted a model put forward by Siever and Davis. Siever and Davis [58] suggested a model that strengthens the role for schizotypal personality traits in schizophrenia. Briefly, they suggested that individuals with elevated schizotypal traits and patients with schizophrenia share a temporal lobe deficit, which is compensated for by lateral prefrontal overactivation in schizotypy, but not in schizophrenia. Nonliteral language comprehension highly relies on frontotemporal interaction [20]; moreover, underactivation in left prefrontal regions has been reported for another type of nonliteral language, metaphors, in schizophrenia [51], [59]. Therefore, language paradigms represent good paradigms to test the hypothesis. Indeed, in our previous publication, we showed that higher degrees of schizotypal personality traits in a non-clinical population resulted in reduced lateral temporal activation, but increased left lateral prefrontal activation, as detected using fMRI [19]. Following Siever and Davis [58], we hypothesize reduced activation in both these regions in schizophrenia.

To our knowledge, no fMRI studies on irony comprehension in schizophrenia have been reported. The aim of this work is to provide the first insights into the functional neuroanatomy of irony comprehension in schizophrenia using fMRI. Our hypotheses for activation abnormalities in schizophrenia are based on the functional models outlined above. We hypothesize a functional deficit in the brain fronto-temporal semantic language system in both cerebral hemispheres. Based on our previous study in non-clinical schizotypal individuals, we think that the strength of the deficit will correlate with the degree of schizotypal traits. Further, we expect that as an alternative, or in addition to, the abnormalities in the frontotemporal system, functional deficit will be observed in TOM regions, including the medial prefrontal cortex and the temporoparietal junction, both of which play a role in irony comprehension in healthy subjects [20]. Prosody may likewise interact with the functional deficits in schizophrenia. Thus, in this investigation, we chose a prosody-free task to limit influencing factors. Deficits in both the medial prefrontal and temporal lobe systems are expected to mirror the findings for irony comprehension in autism [43], [60]. Further, we hypothesize that compensatory activation (fMRI signals greater in schizophrenia patients versus controls), equivalent to literal language perception in schizophrenia [56], may occur in brain regions adjacent to the classical semantic system, such as the premotor cortex.

## Materials and Methods

### Subjects

This study included 15 right-handed probands with DSM-IV schizophrenia and 15 healthy control subjects matched for age, years of education, and verbal intelligence [61]. All study participants were female. The recruitment process and results for the control group were published previously in detail [19]. Patients were recruited from the Department of Psychiatry, University of Tübingen, Germany. All patients were acute or subacute inpatients, were native German speakers, had no other past or present medical illness, and had sufficient reading skills. All patients were on stable medication, mainly with atypical antipsychotics (mean dosis [62] 516 (SD: 237) chlorpromazine equivalents). Among the 15 patients, four patients showed concretism. Among the 15 patients, four patients showed concretism. Schizotypal personality traits (SPQ total score) showed a range between 14 and 70 in patients (range 1–44 in controls [19]). Further group characteristics are shown in table 1.

**Table 1:** Clinical and sociodemographic characteristics of patient and control group.

	patients	control subjects	significance
	n = 15	n = 15	p
age (years)	28.1	32.9	0.12
years of fulltime education	15.9	14.0	0.11
verbal intelligence (score)^1^	31.6	30.2	0.21
HAWIE picture sequencing test^2^	30.7	32.8	0.71
CPT	0.3	1.7	0.04
SPQ cognitive perceptual^3^	7.3	23.1	<0.001
SPQ interpersonal^3^	3.6	14.6	<0.001
SPQ total	14.6	38.4	<0.001
SAPS total score	33.0		
SAPS hallucinations	6.9		
SAPS delusions	17.5		
SANS total score	29.3		
PANSS total score	69.9		
PANSS positive	17.4		
PANSS negative	16.0		
PANSS general	36.5		
Global Assessment of Functioning Scale	39.0		
Chlorpromazine equivalents^4^	516.0		

1 Multiple choice vocabulary test [61].

2 Subtest 2 from [72].

3 As definded by [63].

4 As definded by [62].

### Procedure

The study was approved by the local ethical committee (University of Tuebingen, Germany). First, all subjects received complete information about the study and ability to consent was ensured in an interview with an experienced psychiatrist (A.R.). Afterwards, subjects underwent a practice session with stimuli not used in the experiment and provided written informed consent. Then, the subjects completed the schizotypal personality questionnaire (German version, [63]) and underwent functional magnetic resonance imaging. During the fMRI scanning procedure, subjects lay supine in the MR-scanner, their head secured by foam rubber to minimize movement artefacts. Stimuli were presented as whole sentences visually on a translucent screen viewed by the subjects via a mirror. To reduce the difficulty of the task, the context scenarios were additionally presented acoustically using a tape-recorded version with a female voice. To avoid influences of an ironic tone of voice on brain activation [43], [64], only visual presentation was used in the case of target sentences. During the scanning session, task instruction was to attend to the stimuli and assess if intention of the target sentences was ironic or not. However, to avoid effects of motor response on brain activation, no motor response was requested. Instead, subjects read all sentences silently and performed an attention task during which they pressed a button with their right index finger any time a particular picture appeared on the screen. Stimulus sequence was unforeseeable for the subject and optimised using optseq software (http://surfer.nmr.mgh.harvard.edu/optseq/).

### Experimental Stimuli

A set of 56 German stimuli, each consisting of context scenarios and target sentences, was used in the experiment. Exact description of the stimuli and evaluation process was given previously [19], [57]. In brief, the context scenarios consisted of 2 sentences (8–12 words), each with 2 protagonists. The target sentences always consisted of 1 statement made by one of the protagonists and had, in the context, either an ironic or a literal meaning. The number of words and sentences, grammatical complexity, and word frequency were counterbalanced between literal, ironic, and meaningless context scenarios. Corresponding literal and ironic target sentences were identical.

### Functional MRI Acquisition

Imaging was performed on a 3-T Scanner (Siemens,TIM TRIO). Functional images were acquired with an echoplanar image sequence which is sensitive to BOLD-contrast (TE 40 ms, TR 2 s, 32 slices, slice thickness 3 mm, gap 1 mm, FoV 192×192 mm^2^, pixel size 3×3 mm^2^). One run consisting of 390 volumes was acquired during the experiment. After the functional task, structural images of the whole brain were acquired using a T1-weighted MPRAGE sequence (TR 2200 ms, TI 900 ms, TE 2.92 ms, voxel size 1×1×1 mm^3^).

### Data Analysis

First level processing parameters for the imaging data were identical with [19]. For image processing and all statistical analysis, SPM5 (Wellcome Department of Imaging Neuroscience, London) was used. The functional images of each subject were slice time corrected to the middle slice and were corrected for motion and realigned by using the first scan of the block as reference. T1 anatomical images were coregistered to the mean of the functional scans and spatial normalized to the MNI space by the combined segmentation, bias correction and spatial normalization tool in SPM5. The calculated nonlinear transformation was applied to all functional images. Finally, the functional images were smoothed with an 8-mm full-width, half-maximum (FWHM) Gaussian filter. A general linear model (GLM) was constructed for each participant to analyze the hemodynamic response function. In each GLM, regressors were generated by convolving a box car function with the hemodynamic function. Separate regressors were used to model the hemodynamic responses during presentation of target textoids, ironic sentences, literal sentences and the visual baseline condition. Moreover, a high-pass filter (1/128 Hz) was applied to remove low-frequency drifts.

For each subject, several T-Test contrasts were calculated separately for (1) the ironic target sentences (“irony”) and (2) literal target sentences (“literal”) conditions versus visual baseline and (3) ironic versus literal target sentences. Random effects analyses on group level were calculated for each of these contrasts. Then, between group comparisons were calculated for each of these contrasts using two sample T-Tests.

### Effect of Psychopathology and Schizotypal Personality Traits on Brain Activation

To investigate the influence of psychometric schizotypy and psychopathology on brain activation, simple regression analyses of SPM data were applied. In this type of analysis, each single voxel in the brain is individually examined with respect to whether the size of the BOLD response is correlated with a variable over subjects.

Effects of schizotypal personality traits were calculated using the total score of the schizotypal personality questionnaire ([65], German version [63]) as regressor. These analyses were calculated for all participants together, following the rationale that schizotypal traits represent a continuum [66], [67], [68]. The result of this analysis is a brain map, which depicts the voxels with which there is a significant correlation between the variables. Because of the exploratory character of our pilot-study, we chose a liberal threshold of p<0.001 uncorrected and an extent threshold of 5 voxels for these analyses. In each analysis, separate tests were performed to detect positive correlation (that means the higher the score of the individual score of the study subject, the stronger the BOLD response) and negative correlation (the higher the score of the individual subject, the weaker the BOLD response) were calculated.

Effects of psychopathological parameters were calculated within the schizophrenia group. Due to computer hardware failure, psychopathological data for 2 patients was lost. Therefore, regression analyses were performed using data from only 13 patients. In separate subanalyses, SAPS (positive symptoms, [69]) total score, SANS (negative symptoms, [70]) total score and SAPS formal thought disorder subscale were used as regressors.

### Off-line Testing

After fMRI, the subjects performed an off-line test to detect error rate. In this test, the subjects indicated by button press whether the target sentence has ironic, literal, or meaningless content [19], [57]. Subjects were seated in front of a computer screen. A total of 54 stimuli was used for this experiment (22 ironic and 22 literal textoids identical to the fMRI experiment, 10 textoids with similar structure and content followed by a nonsense statement by one of the two protagonists). The task was to indicate by pressing one out of three buttons whether the target sentence was in this context most likely ironic, literally meaningful, or meaningless. Afterwards, subjects completed a short test battery (verbal intelligence [61], digit span [71], subtest picture sequencing from the HAWIE-R intelligence test [72]). Then, subjects were clinically assessed (by A.R.) using the SAPS [69], SANS [70] and PANSS [73].

## Results

Results of the attention task inside the MR scanner indicate good performance with no significant difference between patients and controls (mean error rate 0.1 errors in healthy controls, 0.8 errors in schizophrenia, p = 0.33).

### Off-line Data

Immediately after the fMRI session, subjects completed the off-line irony test. Off-line performance in the irony comprehension task showed substantial performance in both control subjects (mean 96.3% correct answers, SD 3.4) and schizophrenia (85.3% correct, SD 15.3), however with a significant difference (p = 0.02, ANOVA). A significant correlation was found between psychometric schizotypy (total score of the schizotypal personality questionaire) and and percentage of correct responses (r = −0.55, p = 0.004).

### Brain Activation

Main effects for reading priming sentences, ironic targets, and literal targets showed robust activations in a predominantly left lateralized network including visual cortices, temporal lobe, and prefrontal cortex in the study participants.

### Differences between Control and Patient Group

**Figure 1:**
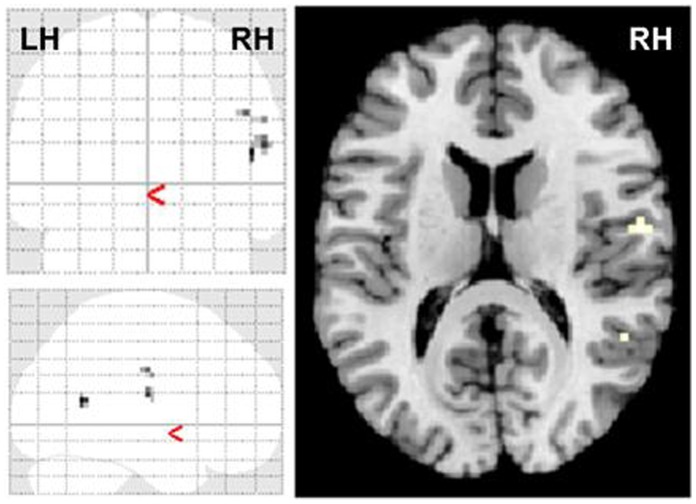
Group comparison healthy controls>schizophrenia for ironic>literal target sentences. p<0.001, ext. 5 voxels. Differences are present in the right hemisphere middle temporal gyrus, rolandic operculum and postcentral gyrus. The opposite contrast (patients>controls) showed no activated clusters.

**Figure 2:**
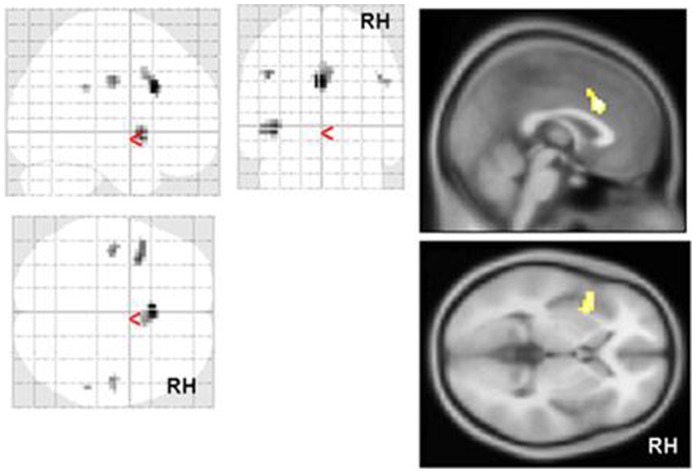
Group comparison healthy controls>schizophrenia for ironic sentences>visual baseline. p<0.001, ext. 5 voxels. Strongest maxima in the posterior part of the anterior cingulate (ACC) and the LH anterior insula. See as well supplemental figure S1.

Results for differential contrasts between control subjects and schizophrenia patients are shown in table 2, figure 1, figure 2 and figure S1.

**Table 2:** Group comparison between healthy controls and schizophrenia. p<0.001, ext. 5 voxels.

region	hemisphere	extent	MNIcoordinates	z
ironic sentences>literal target sentences				
controls >patients				
middle temporal gyrus	RH	5	51 −54 15	3.39
rolandic operculum	RH	8	57 −12 18	3.29
postcentral gyrus	RH	6	48 −12 33	3.25
	RH		57 −9 30	3.19
patients>controls				
No activated clusters				
ironic sentences>visual baseline				
controls >patients				
anterior/middle cingulate gyrus	LH	60	−3 18 27	4.02
insula	LH	41	−36 9 0	3.75
	LH		−45 12 0	3.55
postcentral gyrus	LH	15	−42 −12 33	3.59
postcentral gyrus	RH	11	48 −15 33	3.44
supramarginal gyrus	RH	5	54 −36 30	3.39
patients>controls				
parahippocampal gyrus	LH	26	−24 −36 −12	4.40
brainstem	LH	5	−6 −27 −9	3.74
fusiform gyrus	RH	6	24 −39 −15	3.70
Literal target sentences>visual baseline				
controls >patients				
no activated clusters				
patients>controls				
no activated clusters				

In brief, differential contrasts between ironic and literal target sentences showed diminished activation in right hemisphere temporal and parietal regions in schizophrenia. Further, during processing of ironic, but not literal, target sentences, BOLD response in schizophrenia was decreased in a network including the posterior medial prefrontal cortex (MNI maximum at −3 18 27, Brodmann area 32/24) and left hemisphere (LH) insula. In the reverse contrast (schizophrenia>controls) patients showed enhanced BOLD response in the posterior temporal lobe bilaterally (table 2). Again, this difference was only detectable for ironic, but not literal target sentences.

### Influence of Schizotypal Personality Traits on Brain Activation

**Figure 3:**
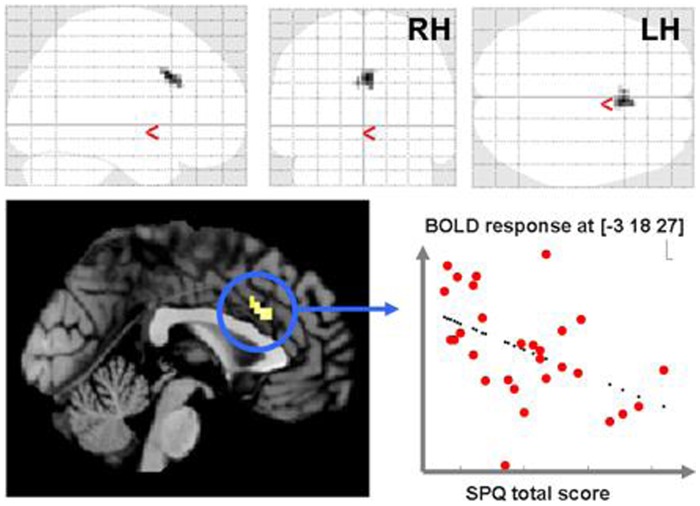
negative correlation between BOLD response at MNI [−3 18 27] and SPQ total score. ACC activation in the cluster with strongest underactivation relative to controls (see figure 2) shows negative correlation with the schizotypal personality questionaire total score. Ironic sentences>visual baseline. For illustrative purposes, threshold of p<0.005, ext. 10 voxels are used.

Influences of schizotypal personality traits, as measured with the total score of the SPQ [63] were calculated across all study participants. As positive and negative correlations indicate different processes, they were calculated separately. A negative correlation was found, that is the higher the degree of psychometric schizotypy, the lesser the BOLD response, in the posterior medial prefrontal cortex (anterior cingulate, MNI x, y, z: 3, 18, 33; z = 3.34) for reading ironic sentences>visual baseline in the same regions previously found underactivated in schizophrenia patients in the differential contrast (figure 3). Reading ironic sentences>visual baseline contrasts showed no positive correlation with the SPQ total score. Further, the contrasts for ironic>literal target sentences or literal sentences>visual baseline did not show significant correlations (neither positive nor negative).

Results from further correlations between cognitive-perceptual and interpersonal subscales of the German schizotypal personality questionaire are shown in table S4. We could not investigate an association with the disorganised factor since, in contrast to the original version, the German version of the SPQ has no disorganised factor [74], [75]. Within the schizophrenia group, there was no significant correlation between the SPQ and PANSS [73] total score (r = 0,08; p = 0,79) or the global assessment of functioning score (r = −0,17; p = 0,58).

### Influence of Psychopathology within the Patient Group

Correlations with symptom dimensions within the patient group were detectable in both cerebral hemispheres are shown in table 3.

**Table 3:** Correlation analysis between fMRI signal during irony comprehension and psychopathology dimensions.

	Positive correlation					Negative correlation				
	region	hemisphere	size	MNI	z	region	hemisphere	size	MNI	z
**Ironic sentences>literal target sentences**										
positive symptoms (SAPS total score)	no activated clusters					insula/superiortemporal gyrus	RH	21	60 −27 0	3.56
Thought disorder (SAPS Thought disorder)	no activated clusters					no activated clusters				
negative symptoms (SANS total score)	no activated clusters					no activated clusters				
**Ironic sentences>visual baseline**										
positive symptoms (SAPS total score)	inferior frontal gyrus	LH	6	−42 30 12	3.59	no activated clustersno activated clusters				
Thought disorder (SAPS Thought disorder)	posterior cingulate	LH	21	−6 −54 6	3.71					
		LH		−12 −48 3	3.62					
	Thalamus	LH	7	−21 −30–3	3.69					
	superior temporalgyrus	RH	7	60 −27 0	3.56					
negative symptoms (SANS total score)	middle temporalgyrus	LH	18	−54 −42 −6	3.51	rolandic operculum	RH	5	57 −6 12	4.45
				−45 −39 −6	3.44	corpus callosum	LH	30	−9 6 24	3.97
							LH		−6 −3 30	3.44
							LH		−18 12 24	3.11
						middle temporalgyrus	RH	8	51 −57 9	3.50
							RH		45 −60 15	3.39
						frontal lobe	LH	7	−24 33 15	3.49
						cerebellum	LH	7	−3 −42 −30	3.46
						corpus callosum	RH	6	6 18 18	3.41
**Literal target sentences>visual baseline**										
positive symptoms (SAPS total score)	no activated clusters					no activated clusters				
Thought disorder (SAPS Thought disorder)	no activated clusters					no activated clusters				
negative symptoms (SANS total score)	inferior frontalgyrus	RH	10	39 27 −15	4.05	precuneus	RH	5	6 −72 33	3.74

Correlations are shown within the schizophrenia group (n = 13). A negative correlation indicates that the higher the degree of psychopathology, the lower is the BOLD response. p<0.001, ext. 5 voxels.

## Discussion

We investigated the comprehension of ironic and literal text vignettes in female patients with schizophrenia by using event-related fMRI. In a prosody-free reading task, subjects processed text vignettes that ended in either a literal or an ironic statement made by one of the protagonists. As expected, we were able to detect robust differences between patients and subjects in a control group, which was matched for age and educational level (control group results previously published [19]). Differences were detectable both for ironic versus literal, as well as for ironic sentences versus visual baseline, between the groups. However, no differences were detected for reading literal target sentences vs. visual baseline. This lack of difference is in line with a number of fMRI studies on literal language in schizophrenia [56].

In this pilot study, we had several hypotheses concerning the results, which were partially confirmed. Most investigations in healthy subjects (see [20]), as well as those with brain lesions (see [19]), indicate that both cerebral hemispheres are involved in the comprehension of ironic remarks. In healthy individuals, the contribution of the right hemisphere seems to be more prominent for irony than for literal language [20], [21], [22], [33]. Based on these and other studies, we hypothesised that impairment in irony comprehension in both schizophrenia and autism may be caused by right hemisphere dysfunction [10], [76]. However, our results have been mixed in terms of cerebral lateralization. Indeed, contrasts for ironic vs. literal (Table 2) indicate a deficit in the right cerebral hemisphere; however, contrasts between ironic target sentences vs. visual baseline and correlations with symptomatology (Table 3) indicate that the left hemisphere also contributes to the deficit.

Our main hypothesis was that dysfunction of the brain’s frontotemporal language system may be crucial in the pathophysiology of the difficulties experienced by patients with schizophrenia in interpreting ironic remarks, and that schizotypal personality traits might mediate the magnitude of these difficulties. As expected, patients showed attenuated activation in the RH middle temporal gyrus (table 2, figure S1).

Moreover, BOLD response in the RH temporal lobe showed correlations with both positive and negative symptoms (table 3). The right hemisphere temporal lobe is part of the brains semantic system [20], [77]. Our finding of decreased activation during a language task in schizophrenia therefore supports other evidence of impairment of this system in schizophrenia [47], [56], [78]. However, in contrast to our expectation [19], and in contrast to other fMRI findings for figurative language in schizophrenia [51], [59], there was no activation difference in the left inferior frontal gyrus.

The cognitive model for delusions by Salvatore et al. [27], [79] assumes that ambiguous intersubjective interactions, such as ironic remarks, are relevant for the development of positive symptoms in schizophrenia and may be caused by mirror neuron dysfunction. The brain regions typically associated with mirror neurons are the ventral premotor cortex and the inferior parietal lobule [80], and these regions did *not* show the most prominent differences between patients and controls in our study, although they are known to be involved in irony comprehension in healthy subjects [19], [20]. However, the human mirror neuron system may extend into other brains regions as well [80].

The most prominent differences between healthy controls and patients with schizophrenia have been found in the posterior medial prefrontal cortex, as well as the LH insula, RH middle temporal gyrus, and bilateral postcentral gyrus (Table 2). As hypothesised, medial prefrontal brain activation was attenuated in patients with schizophrenia (table 2, table S1). However, activation was localised more posterior than that suggested by the “classical” theory of mind regions [81], [82] in the dorsal part of the anterior cingulate and more posterior than activations found in previous studies on irony comprehension (see [20]). This more posterior part of the anterior cingulate is thought to play a role in conflict monitoring and action monitoring [81] and in making judgements about the external world [82]. Therefore, activation in this part might represent a correlate of the difficulties of patients with schizophrenia to simulate the social situation during the decision process and to determine if a sentence is ironic or not. The medial prefrontal cortex is also ascribed the function to suppress the incorrect alternative literal meaning during comprehension of nonliteral stimuli [20], [83], so attenuated activation in this area could possibly reflect the tendency of patients with schizophrenia to literally interpret nonliteral stimuli (“concretism”).

In the present study, the degree of attenuation of the BOLD response in this region correlated with psychometric measures of schizotypy across all subjects, i.e., the higher the SPQ score, the lesser the BOLD response exhibited by the subject during the processing of ironic sentences, irrespective of diagnostic group. Thus, our results further strengthen previous assumptions of a continuum between schizotypal traits in nonclinical subjects and symptoms manifested in patients with schizophrenia [67], [68]. Our data are also compatible with previously made assumptions according to which the interaction between medial prefrontal and lateral temporal brain areas might be determined by the magnitude of schizotypy expression and other subclinical psychotic symptoms. For example, based on their research on schizotypy in nonclinical adolescent subjects, Lagioa et al. [84] suggested that schizotypy is associated with “inefficient connectivity” between medial prefrontal and language areas [84]. Similarly, Brent et al. [85] suggested, on the basis of fMRI research on subclinical psychotic symptoms in their nonclinical population, that “aberrant connectivity” between frontal and lateral temporal areas may play a role in the pathophysiology of psychosis.

Another region found to be underactivated in schizophrenia was the LH insula. The insula is involved in perceptual decision making [86], [87], [88] and in the comprehension of irony [89] and other nonliteral stimuli in healthy subjects [20], [90], [91]. The insula shows structural and functional abnormalities in schizophrenia [92]. Underactivation of the insula in fMRI studies has been previously reported during social cognition tasks [93] and language comprehension on sentence level in schizophrenia [56], [59], [94].

Patients showed stronger BOLD response than controls in the left hemisphere parahippocampal gyrus. This region frequently shows activation during the comprehension of nonliteral stimuli, which could possibly represent ambiguity processing and analyzing/ascribing emotional connotation to nonliteral stimuli [20]. It could also represent “integrating context” [90], [95] or “recognizing the importance of social cues” during irony comprehension [90]. A growing body of evidence suggests that the parahippocampal gyrus may play a significant role in schizophrenia. For example, in fMRI studies, aberrant activation of the parahippocampal gyrus has been shown to be associated with positive symptoms in patients with schizophrenia [96], [97], [98], [99]. Rankin et al. [90] speculated that defective “top-down influence on the parahippocampal gyrus” from the dorsomedial prefrontal and insular cortex may play a role in the pathophysiology of defective sarcasm detection in neurodegenerative diseases, arguing that these brain regions are functionally interconnected. The same mechanism may also play a role in the pathophysiology of defective irony comprehension in schizophrenia, as we found impaired activation in the dorsomedial prefrontal cortex and insula, and increased activation in parahippocampal regions.

### Limitations

We are aware of several limitations in our study. First, the number of subjects is rather low, especially when considering that correlation analyses were performed. This gives our investigation more of the character of a pilot study. Future research is therefore needed to confirm the reliability of the findings in a larger sample. On the other hand, stable and replicable correlations with personality traits and psychopathology have been shown in studies with roughly the same number of subjects [100], [101], [102]. Furthermore, conference proceedings with data from an additional fMRI study on irony comprehension in schizophrenia confirm the underactivation of the insula in schizophrenia [103].

Affective connotation is a further limitation. In our task, ironic statements were predominantly negative. However, irony with positive connotations may have different neural correlates [104]. Furthermore, we studied only female individuals. This point is of possible importance because gender differences have been reported for irony comprehension [105], [106]. Furthermore, gender differences have been reported for schizotypy [107] and schizophrenia [108]. Future research is therefore encouraged to evaluate how irony comprehension in schizophrenia interacts with gender [109].

Previous fMRI studies have showed that irony comprehension may be related to motor cortex function. To avoid confounding with button press or motor response, our subjects had to indicate whether the sentence was ironic or not only in the offline task. Thus, we cannot be certain that subjects performed the task in the scanner correctly. Nevertheless, good performance in the attention task, the quality of movement parameters during the imaging session, and stable activation in the brain’s language system in each individual subject do indicate that subjects complied with the instructions.

Several limitations relate to the nature of our irony comprehension task. Irony is a complex phenomenon and can have various linguistic forms. In our irony comprehension test, most stimuli characteristics were paralleled between ironic and literal text vignettes. In the target sentences, a protagonist made an ironic or literal statement. Our task was explicit (i.e., subjects were aware that ironic utterances may occur during the task), and ironic statements were made concerning others (not relating to the study participants). It is possible that patients with schizophrenia might show aberrant responses when dealing with issues pertaining to them personally, but this was not the case here. In a more general sense, it is very likely that social cognition in a real world setting might be different [19], [110], [111]. In interpersonal communication that is not in written form (which was used here), it is postulated that subjects include information from facial affect, prosody, larger context, information about the speaker, momentary affect, general world knowledge, and other factors when trying to decide whether a statement is ironic or not. All of these factors have been shown to undergo altered processing in schizophrenia, and aberrant activation of various brain regions has been confirmed in functional imaging studies in connection with most of these factors in patients with schizophrenia. Thus, it is obvious that our study represents only the beginning in this investigation, and future research must clarify how these other factors interrelate with schizophrenic psychopathology during irony appreciation.

## Supporting Information

Figure S1 **Group comparison between healthy controls and schizophrenia for ironic sentences>visual baseline. Slice view.** p<0.001, ext. 5 voxels. Controls>schizophrenia activation is marked red, Strongest maxima in the posterior part of the anterior cingulate (ACC) and the LH anterior insula. Schizophrenia>controls is marked blue, strongest activation is in the LH parahippocampal gyrus.
(PSD)

Table S1 **Correlation analysis between fMRI signal during irony comprehension and schizotypal personality traits.** Correlations are shown across all study participants independent of diagnosis. p<0.001, ext. 5 voxels. Table shows correlations with “interpersonal” and “cognitive perceptual” subscales of the schizotypal personality questionnaire, German version [67]. A negative correlation indicates that the higher the degree of psychometric schizotypy, the lower is the BOLD response. Total score of the schizotypal personality questionnaire showed negative correlation in the posterior medial prefrontal cortex (MNI 3 18 33, z = 3,34, extent 5 voxel) for reading ironic sentences>visual baseline), all other correlations were not significant.
(DOC)
