# Supplementary material for: Isn’t it ironic? Neural Correlates of Irony Comprehension in Schizophrenia
Source: PLoS One. 2013 Sep 10;8(9):e74224. doi: 10.1371/journal.pone.0074224 (PMC3769349; doi:10.1371/journal.pone.0074224)
Supplement: Table S1 — Correlation analysis between fMRI signal during irony comprehension and schizotypal personality traits. Correlations are shown across all study participants independent of diagnosis. p<0.001, ext. 5 voxels. Table shows correlations with “interpersonal” and “cognitive perceptual” subscales of the schizotypal personality questionnaire, German version [67]. A negative correlation indicates that the higher the degree of psychometric schizotypy, the lower is the BOLD response. Total score of the schizotypal personality questionnaire showed negative correlation in the posterior medial prefrontal cortex (MNI 3 18 33, z = 3,34, extent 5 voxel) for reading ironic sentences>visual baseline), all other correlations were not significant. (DOC) [file pone.0074224.s002.doc]

**Supplemental Table S1**

**Correlation analysis between fMRI signal during irony comprehension and schizotypal personality traits.** Correlations are shown across all study participants independent of diagnosis. p< 0.001, ext. 5 voxels. Table shows correlations with “interpersonal” and “cognitive perceptual” subscales of the schizotypal personality questionnaire, German version [67]. A negative correlation indicates that the higher the degree of psychometric schizotypy, the lower is the BOLD response. Total score of the schizotypal personality questionnaire showed negative correlation in the posterior medial prefrontal cortex (MNI 3 18 33, z=3,34, extent 5 voxel) for reading ironic sentences > visual baseline), all other correlations were not significant.

**Table S1**a Interpersonal subscale

| Positive correlation | | | | | Negative correlation | | | | |
| --- | --- | --- | --- | --- | --- | --- | --- | --- | --- |
| region | hemisphere | size | MNI | z | region | hemisphere | size | MNI | z |
| **Ironic sentences > literal target sentences** | | | | | | | | | |
| corpus callosum frontal lobe | LH | 7 | -6 21 0 | 3.77 | no activated clusters | | | | |
| **ironic sentences > visual baseline** | | | | | | | | | |
| middle temporal gyrus | LH | 8 | -51 -42 -3 | 3.77 | rolandic operculum | RH | 15 | 57 -6 12 | 3.67 |
| **literal sentences > visual baseline** | | | | | | | | | |
| brainstem |  | 5 | -3 -21 -21 | 4.21 | no activated clusters | | | | |
| middle temporal gyrus | LH | 14 | -45 -48 18 | 4.14 |

**Table S1**b cognitive perceptual subscale

| Positive correlation | | | | | Negative correlation | | | | |
| --- | --- | --- | --- | --- | --- | --- | --- | --- | --- |
| region | hemisphere | size | MNI | z | region | hemisphere | size | MNI | z |
| **Ironic sentences > literal target sentences** | | | | | | | | | |
| no activated clusters | | | | | no activated clusters | | | | |
| **ironic sentences > visual baseline** | | | | | | | | | |
| claustrum | LH | 8 | -30 -21 9 | 3.35 | no activated clusters | | | | |
| parahippocampal gyrus | RH | 5 | 30 -30 -9 | 3.31 |
| literal sentences > visual baseline | | | | | | | | | |
| no activated clusters | | | | | no activated clusters | | | | |
